# From the Experience of Interactivity and Entertainment to Lower Intention to Smoke: A Randomized Controlled Trial and Path Analysis of a Web-Based Smoking Prevention Program for Adolescents

**DOI:** 10.2196/jmir.7174

**Published:** 2017-02-16

**Authors:** Georges Elias Khalil, Hua Wang, Karen Sue Calabro, Natasha Mitra, Ross Shegog, Alexander V Prokhorov

**Affiliations:** ^1^ MD Anderson Cancer Center Department of Behavioral Sciences University of Texas Houston, TX United States; ^2^ University at Buffalo Department of Communication Buffalo, NY United States; ^3^ Health Science Center at San Antonio School of Public Health University of Texas San Antonio, TX United States; ^4^ Health Science Center at Houston Center for Health Promotion and Prevention Research University of Texas Houston, TX United States

**Keywords:** smoking prevention, intention to smoke, adolescent, Web-based intervention, interactivity, entertainment, emotions, presence, experience

## Abstract

**Background:**

Web-based programs for smoking prevention are being increasingly used with some success among adolescents. However, little is known about the mechanisms that link the experience of such programs to intended nicotine or tobacco control outcomes.

**Objective:**

Based on the experiential learning theory and extended elaboration likelihood model, this study aimed to evaluate the impact of a Web-based intervention, A Smoking Prevention Interactive Experience (ASPIRE), on adolescents’ intention to smoke, while considering the experience of interactivity and entertainment as predictors of reduced intention to smoke, under a transitional user experience model.

**Methods:**

A total of 101 adolescents were recruited from after-school programs, provided consent, screened, and randomized in a single-blinded format to 1 of 2 conditions: the full ASPIRE program as the experimental condition (n=50) or an online , text-based version of ASPIRE as the control condition (n=51). Data were collected at baseline and immediate follow-up. Repeated-measures mixed-effects models and path analyses were conducted.

**Results:**

A total of 82 participants completed the study and were included in the analysis. Participants in the experimental condition were more likely to show a decrease in their intention to smoke than those in the control condition (beta=−0.18, *P*=.008). Perceived interactivity (beta=−0.27, *P*=.004) and entertainment (beta=−0.20, *P*=.04) were each associated with a decrease in intention to smoke independently. Results of path analyses indicated that perceived interactivity and perceived entertainment mediated the relationship between ASPIRE use and emotional involvement. Furthermore, perceived presence mediated the relationship between perceived interactivity and emotional involvement. There was a direct relationship between perceived entertainment and emotional involvement. Emotional involvement predicted a decrease in intention to smoke (beta=−0.16, *P*=.04).

**Conclusions:**

Adolescents’ experience of interactivity and entertainment contributed to the expected outcome of lower intention to smoke. Also, emphasis needs to be placed on the emotional experience during Web-based interventions in order to maximize reductions in smoking intentions. Going beyond mere evaluation of the effectiveness of a Web-based smoking prevention program, this study contributes to the understanding of adolescents’ psychological experience and its effect on their intention to smoke. With the results of this study, researchers can work to (1) enhance the experience of interactivity and entertainment and (2) amplify concepts of media effects (eg, presence and emotional involvement) in order to better reach health behavior outcomes.

**Trial Registration:**

Clinicaltrials.gov NCT02469779; https://clinicaltrials.gov/ct2/show/NCT02469779 (Archived by WebCite at http://www.webcitation.org/6nxyZVOf0)

## Introduction

Tobacco smoking remains the most preventable cause of death in the world [1]. However, more than 20% of adolescents in the United States have smoked at least one cigarette [2], and their use of new and emerging nicotine and tobacco products has increased [2-5]. Approximately 80% of adult daily tobacco smokers begin using before the age of 18 years and become addicted during adolescence [1,6]. This suggests the need for continuous efforts to prevent smoking among adolescents. In particular, Web-based programs are increasingly used to improve tobacco risk communication and prevent smoking [7-9].

While several Web-based programs have shown success in delaying smoking initiation among adolescents [10-14], little is known about adolescents’ experience with such programs. This is evident from meta-analyses and reviews on the evaluation of smoking prevention programs [10-12,15]. First, evaluation studies tend to focus solely on *health outcomes* (eg, intention to smoke and smoking initiation behavior [10-12,15]) without examining psychological media effects that may explain change in outcomes. The investigation of media effects related to one’s experience during program use may shed light on how a Web-based program achieves success. Second, cognitive theories dominate program evaluations [11,16], leaving aside the role of emotional involvement in driving successful outcomes. In particular, studying cognitive processes of behavior change alone can be limiting, especially with adolescents whose decision making is often dependent upon their emotions.

To fill in these gaps, it is important to better understand the processes by which Web-based interventions work to reach health outcomes. Going beyond a mere evaluation that explains “whether” a program works, it is crucial to investigate “how” a program works to be successful. This is done by studying the underlying mechanism that delineates one’s experience of a Web-based intervention.

As a result, in this study, we aimed to test associations that link participation in a Web-based program to changes in a health outcome. We achieved this aim in the context of a Web-based intervention called *A Smoking Prevention Interactive Experience* (ASPIRE) [17-20]. Web-based interventions such as ASPIRE make use of two main design elements: interactivity and entertainment [20]. *Interactivity* refers to a technology design that allows for a two-way communication between adolescents and the program (eg, wide range of options, ability to explore virtual environments, and connect with other users) [21]. *Entertainment* refers to a design that is expected to drive enjoyment and gratification (eg, comedy, humor, fear appeal, and game play) [22-25]. In ASPIRE, features of interactivity and entertainment are applied through entertaining videos that intertwine with interactive and entertaining activities relevant to smoking-related issues. Several associations that link interactivity and entertainment to health outcomes deserve attention.

With a careful design of interactive and entertaining features, users’ experience of interactivity and entertainment becomes crucial for the success of Web-based programs [26-30]. *Perceived interactivity* [31] refers to the user’s perception of an active two-way communication with the program and a sense of control over online behavior [21,26-29,32]. *Perceived entertainment* [31,33] refers to the users’ beliefs that their experience with the program is enjoyable and entertaining [30,34-36]. Both perceived interactivity and perceived entertainment are built on the idea that interactivity and entertainment are subjective experiences [26-29]. Research has supported a multidimensional perspective of entertainment that is based on the experience of psychological amusement, pleasant atmosphere, and joy [37]. Research has also supported that users must be psychologically involved and build a sense of influence in order to experience interactivity [21].

The effect of the experience of interactivity and entertainment on health outcomes is supported by the experiential learning theory (ELT) [38] and the extended elaboration likelihood model (E-ELM) [39]. According to the ELT, users of interactive applications are able to learn through the exploration of environments. In essence, first-hand exploration fosters curiosity and ultimately facilitates learning. According to the E-ELM, individuals exposed to entertainment programming begin to feel transported into the program environment by (1) becoming immersed in the world of the program and (2) becoming emotionally involved in the program. Ultimately, such transportation drives support for the healthy behavior.

Supportive of the ELT and E-ELM, empirical findings show that users’ experience of interactivity and entertainment gradually drives them toward healthy outcomes. Perceived interactivity [40-44] and entertainment [22-25] have been found to drive attention to media platforms. This attention is expected to create *perceived presence* (ie, individuals’ perception of being present inside the environment of the website [45]). Perceived presence is a key outcome in the context of Web-based interventions because interactivity and entertainment involve the experience of environments that demand attention and immersion. As a result of perceived presence, users of Web-based programs may experience *emotional involvement* (ie, the intensity of felt emotions as a result of the intervention [46-49]). Research has shown that perceived presence is greater in the context of emotional environments, and a greater emotional state is experienced as a result of a higher level of presence [50]. Ultimately, emotional involvement can have an impact on psychological antecedents of nicotine or tobacco use (eg, lowering intention to smoke) through affective persuasion [51]. Such line of research suggests a model by which adolescents move from intervention exposure to changes in tobacco control outcomes.

**Figure 1:**
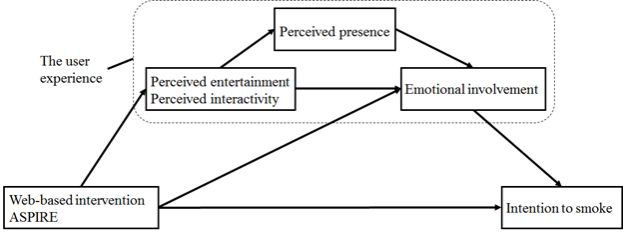
Conceptual model tested in this study. ASPIRE: A Smoking Prevention Interactive Experience.

Although such associations have been examined each on its own, they remain fragmented. As a result, through a short-term randomized controlled trial with ASPIRE, this study developed a user experience model and statistically validated it using path analysis (Figure 1). We hypothesized the following: exposure to ASPIRE is positively related to perceived interactivity and perceived entertainment (hypothesis 1); adolescents who use ASPIRE are more likely to show a decrease in intention to smoke compared with adolescents in a control group (hypothesis 2); adolescents’ perceived interactivity and perceived entertainment during ASPIRE are positively related to a decrease in intention to smoke (hypothesis 3); perceived interactivity and perceived entertainment mediate the relationship between ASPIRE use and emotional involvement (hypothesis 4); perceived presence mediates the relationship between perceived entertainment and emotional involvement (hypothesis 5); perceived presence in the ASPIRE environment mediates the relationship between perceived interactivity and emotional involvement in ASPIRE (hypothesis 6); and emotional involvement is related to a decrease in intention to smoke (hypothesis 7).

## Methods

### Study Design

In order to examine the net benefit of interactivity and entertainment in ASPIRE, this study was conducted post hoc, using data from a 2-arm single-blinded randomized controlled trial with assessments at baseline and immediate follow-up (time × condition). This trial, called ASPIRE Reactions, was conducted in 2014. It is registered at the ClinicalTrials.gov registry (identifier: NCT02469779). Its components adhere to the CONSORT (Consolidated Standards of Reporting Trials) and CONSORT-EHEALTH (Consolidated Standards of Reporting Trials of Electronic and Mobile Health Applications and Online Telehealth) guidelines [52,53].

### ASPIRE and ASPIRE-Control

The trial involved 2 conditions: (1) the ASPIRE program and (2) a control condition without interactivity or entertainment (ASPIRE-control). Study manipulations are presented in Table 1 (see Multimedia Appendix 1). ASPIRE was presented in 4 sessions, 40 minutes each, spread over a period of 1 month [54].

**Table 1:** Manipulations for study conditions.

Main factor and elements		ASPIRE^a^	ASPIRE-control
**Interactivity**			
	Two-way communication	Yes	No
	Control over platform	Yes	No
	Clicking behavior	Yes	No
	Virtual environments	Yes	No
**Entertainment**			
	Narrative or storytelling	Yes	No
	Music; sound effects	Yes	No
	Virtual characters or avatars	Yes	No
Channel		Multimedia (video, audio, and text)	Text only
Content		Facts delivered in a personal context (age-tailored)	Facts only
Involvement		Emotional (peripheral processing of animation and narratives)	Cognitive (central processing of facts)

^a^ASPIRE: A Smoking Prevention Interactive Experience.

ASPIRE features interactivity and entertainment to engage adolescent users through text, animations, videos, and task-oriented activities (Multimedia Appendix 2). Text mainly involves educational facts about tobacco. As part of the videos, users are presented with cartoon animations and testimonies from high school students. ASPIRE's multimodality is also characterized by the presence of activities that allow users to explore health information in a two-dimensional environment. An activity in ASPIRE entails an environment with an expected task. By completing the task, the user gets to uncover health information. For instance, as part of an activity, users attempt to make a virtual character smoke in order to discover the poisons and chemicals that are left in the lungs (Multimedia Appendix 3). Also, ASPIRE allows users to enter a mediated environment in which they can search for clues, click on objects or characters, and listen to health messages [55]. In addition, ASPIRE includes videos that rely on humorous messages, dramatic events, and real-life stories about tobacco consequences. ASPIRE can be accessed online for free [54].

ASPIRE-control was designed to include the same health information presented in ASPIRE but without any features of interactivity or entertainment. In order to design an appropriate control condition, we conducted qualitative content analysis of ASPIRE’s content to identify factual information about tobacco or smoking that is depicted in ASPIRE. Factual information was compiled and summarized to form a text-based document. Following this procedure, the document was fragmented to form a series of paragraphs. The paragraphs presented information in the same order as presented in ASPIRE. In order to control for exposure to online health information, the text was introduced in a mock website that had the same background design as the actual ASPIRE intervention.

### Sample Recruitment and Procedure

A total of 4 after-school programs located in Medicaid-eligible districts of Houston, Texas, were randomly selected for participant recruitment, including the Boys and Girls Clubs (2 sites), the Salvation Army Boys and Girls Clubs (1 site), and the Young Men's Christian Association (YMCA; 1 site). After approval from the program directors, a verbal announcement reached 509 adolescents. Interested adolescents completed child assent and parental permission. We assessed adolescent eligibility through a screening conducted before participation. Inclusionary criteria were as follows: being of ages 12 through 18 years, being a student in a middle school or high school, and being a nonsmoker (have not smoked in the past year, not even one cigarette, cigar, or hookah).

All participants in the final sample obtained parental consent. Recruitment and data collection took a period of 4 months. The institutional review boards for human subjects research at the University of Texas MD Anderson Cancer Center and the University at Buffalo, the State University of New York approved this study.

All participants started their experience with the intervention 3 days after they completed the baseline survey. The principal investigator generated the random allocation sequence. Research assistants assigned enrolled adolescents to groups. We used concealed envelopes to conduct randomization. Participants were not told which intervention was the intervention of interest. In ASPIRE and ASPIRE-control, participants used identical computers and had private space for individual viewing and headphones for noise reduction. A research assistant was available for technical assistance and supervision. At the end of the intervention, participants completed the follow-up survey. Then, 20 randomly selected participants from the ASPIRE group participated in exit interviews (data not included in this paper).

As an ethical consideration, after follow-up assessment, the ASPIRE-control group received information about the actual ASPIRE intervention and ways to access the website. Each participant was offered a US $15 gift card for participation in the study. Participants received giveaway items such as pens, bags, and earphones to complete each survey.

### Measures

All survey measures have been previously tested and validated. All measures were assessed through Web-based closed surveys, in the presence of a research assistant who was only available for technical assistance. We adhered to the Checklist for Reporting Results of Internet E-Surveys (CHERRIES; Multimedia Appendix 4) [56]. At baseline and follow-up, we measured intention to smoke [57,58] as the primary outcome. Intention to smoke, measured using the susceptibility to smoke scale, is the most potent and direct predictor of smoking initiation among adolescents [59,60]. At follow-up, we measured perceived interactivity [61], perceived entertainment [62,63], perceived presence [64], and emotional involvement [65,66]. We measured perceived credibility of message [67] for both conditions as a measure of manipulation check. To obtain a valid assessment of user experience and program effect, we included questions pertaining to potential confounders, such as environmental and social factors, and demographic characteristics that may contribute to smoking intention [68-73]: age, gender, ethnicity, average grade at school, the number of detentions at school, prior knowledge about the effects of smoking [74], number of friends who smoke, frequency of Internet use in hours per day, and skills in Internet use [75]. A detailed description of the main measures, measure derivation references, and Cronbach alpha values are reported in Table 2.

**Table 2:** Survey measures.

Measures		Description	Alpha^a^
**Health outcome**			
	Intention to smoke	Items adapted from the susceptibility to smoke scale [58-60]: “Do you think in the future you might try a cigarette?”; “If one of your best friends were to offer you a cigarette, would you try it?”; and “Do you think you will try smoking some day in the next 5 years?” Items presented on a 5-point Likert scale with answer choices: 5=definitely yes, 4=probably yes, 3=possibly, 2=probably not, and 1=definitely not.	.80
**User experience**			
	Perceived interactivity	Measured with 17 items from Coursaris and Sung [61], such as “I felt that I had control over my surfing experience of the website” and “The website seemed to be effective in getting my feedback.”	.94
	Perceived entertainment	A scale adapted from the work of Cyr and colleagues [62] and Nysveen and colleagues [63], with two dimensions: enjoyment and fulfillment. Examples of statements: “I found my visit to this website entertaining” and “I found my visit to this website fun.”	.92
**Media effect outcomes**			
	Perceived presence	Measured using 5-point Likert scale items such as “While using the website, I had a sense of being in the scenes” and “While using the website, I felt I was visiting the website’s world” [64].	.88
	Emotional involvement	Two items: The first item belongs to the emotional involvement dimension of the transportation concept [65]: “I felt emotionally involved in the website.” The second item asked participants how much they felt emotionally involved in the website [66]. Answer choices ranged from 1=not at all to 10=extremely.	.55^b^
**Covariates**			
	Prior knowledge	21 items tested knowledge about smoking consequences. Participants indicated if they believe such items are actual consequences of smoking by answering “yes” (coded 1 if correct), “no” (coded 1 if correct), or “I do not know” (always coded 0, as incorrect) [74].	-
	Number of friends who smoke	One open-ended question: “How many of your friends smoke?”	-
	Frequency of Internet use	One open-ended question: “How many hours per day do you spend on the internet?”	-
	Number of school detentions	One open-ended question: “How many detentions or suspensions have you received at school?”	-
	School grades	Total grade at school, based on grade point average. Answer choices were A, B, C, D, or F (coded 1, 2, 3, 4, and 5, respectively).	-
	Perceived credibility	Two items such as “In your opinion, how believable was the information presented in ASPIRE?” [67].	

^a^Reliability coefficients with Cronbach alpha were calculated from posttest data, with the exception of measures with data collected at baseline only.

^b^Indicates Pearson correlation between 2 items, instead of Cronbach alpha.

### Statistical Analysis

We conducted power analysis for sample size calculation. In order to conduct mixed-effects models using a target power of 0.95 and an effect size of 0.20 to predict intention to smoke [76] with an alpha value of .05, the estimated sample size is 80 participants (40 participants in each condition). Approximately 100 adolescents were needed to test these hypotheses, with an anticipated completion rate of 80% (80/100).

Statistical analyses were conducted using Stata version 14 (StataCorp LP). Analyses of variance (ANOVAs) and chi-square tests were conducted to capture any baseline differences between the 2 conditions with respect to covariates (eg, demographic characteristics, grades at school, number of detentions, and frequency of Internet use). Then, manipulation checks were conducted to ensure that both conditions are found to provide credible health information. Bonferroni adjustment was performed to guard against type I error in the repeated ANOVAs [77,78].

To test hypothesis 1, one-way ANOVA was conducted to assess whether using ASPIRE is related to perceived interactivity and perceived entertainment. For hypothesis 2, a repeated-measures mixed-effects model was conducted to test change in intention to smoke, in a 2 (condition) × 2 (time) design. ASPIRE effects on outcome trajectories over time were measured by the condition × time interaction term. To test hypothesis 3 on the effects of perceived interactivity and perceived entertainment, 3 repeated-measures mixed-effects models were conducted with intention to smoke as the outcome variable. Model 1 tested perceived interactivity alone, model 2 tested perceived entertainment alone, and model 3 included both as independent variables. For all models, multicollinearity was tested and the Huber-White sandwich estimator was used to correct all variance estimates for heteroskedasticity [79,80]. For each finding, standardized coefficients were computed with their respective *P* values.

Hypotheses 4 through 7 were first examined using mixed-effects models and multiple regression analyses that adjusted for effects of potential confounders, as covariates. These adjustments did not alter primary conclusions (data not shown). To confirm hypotheses 1 through 3 and test for hypotheses 4 through 7 under one model (Figure 1), path analysis was conducted. Path analysis has been regarded as a preferred method of mediation analysis as it allows the control for measurement error and uncovers information on the degree of fit of the entire model [81-86]. Model fit criteria were as follows: (1) a nonsignificant chi-square goodness-of-fit statistic, (2) a comparative fit index (CFI) of .90 or greater, (3) a root mean square error of approximation (RMSEA) less than or equal to .06, and (4) a standardized root mean square residual lower than .08 [87]. Two models were constructed with (1) perceived interactivity and (2) perceived entertainment as drivers of change. For each model, modification indices were examined to give insight into possible structural aspects of model misfit and update the model based on the indices.

## Results

### Participants

Table 3 presents respondents’ sociodemographic characteristics. For the entire sample (N=101), the average age was 13.44 (SD 1.42) years, 43.56% (44/101) were female, and the majority of participants were black or African American (42/101, 41.58%) and Hispanic or Latino (44/101, 43.56%). Approximately 45% (43/101) reported having at least one friend who smokes, and the number of friends who smoke ranged between 1 and 23 (mean 1.96, SD 3.67). Also, 62.38% (63/101) reported using the Internet at least 2 hours per day, and the hours of use ranged between 30 minutes and 16 hours (mean 4.40, SD 3.70). The 2 groups differed with respect to participants’ number of friends who smoke, *F*_1,94_=5.74, *P*=.02. Subsequent analysis controlled for the difference.

**Table 3:** Characteristics of study participants.

Characteristics		ASPIRE^a^ (n=50) n (%)	ASPIRE-control (n=51) n (%)	Total sample (N=101) n (%)	*P* value^b^
**Age range, years**					
	12-13	26 (52)	38 (74.5)	64 (63.2)	.05
	14-15	18 (36)	11 (21.5)	29 (28.7)	
	16-17	6 (12)	2 (3.9)	8 (7.9)	
**Gender**					
	Male	31 (60.7)	27 (52.9)	58 (56.8)	.42
	Female	20 (39.2)	24 (47.0)	44 (43.5)	
**Race or ethnicity**					
	Hispanic or African American	39 (78)	47 (92.1)	86 (85.1)	.05
	Non-Hispanic, non–African American	11 (22)	4 (7.8)	15 (14.8)	
**Educational level of mother**					
	High school or less	16 (32.6)	23 (45.1)	39 (39.0)	.43
	College or more	33 (67.3)	28 (54.9)	61 (61.0)	
**Educational level of father**					
	High school or less	23 (46.9)	29 (61.7)	52 (54.1)	.27
	College or more	26 (53.0)	18 (38.3)	44 (45.8)	
**Educational level of legal guardian**					
	High school or less	9 (24.3)	12 (46.1)	21 (33.3)	.16
	College or more	28 (75.6)	14 (53.8)	42 (66.6)	
**Average school grades**					
	A	37 (72.5)	31 (60.7)	68 (66.6)	.49
	B	12 (23.5)	17 (33.3)	29 (28.4)	
	C	2 (3.9)	2 (3.9)	4 (3.9)	
	D	0 (0.0)	1 (1.9)	1 (0.9)	
Number of school detentions, mean (SD)		1.52 (3.95)	1.69 (2.65)	1.61 (3.34)	.80
Number of friends who smoke, mean (SD)		1.06 (1.98)	2.82 (4.62)	1.96 (3.67)	.02
Prior knowledge of smoking effects, mean (SD)		14.04 (3.80)	12.94 (4.03)	13.49 (3.94)	.16
Prior intention to smoke, mean (SD)		1.43 (0.65)	1.56 (0.72)	1.50 (0.68)	.36
Frequency of Internet use, mean (SD)		3.77 (3.48)	5.01 (3.83)	4.40 (3.70)	.10

^a^ASPIRE: A Smoking Prevention Interactive Experience.

^b^Significance testing with chi-square test for the categorical variables (ie, age, gender, race or ethnicity, educational level, and school grades) and analysis of variance for the continuous variables. Missing values are not presented in this table.

### Attrition

**Figure 2:**
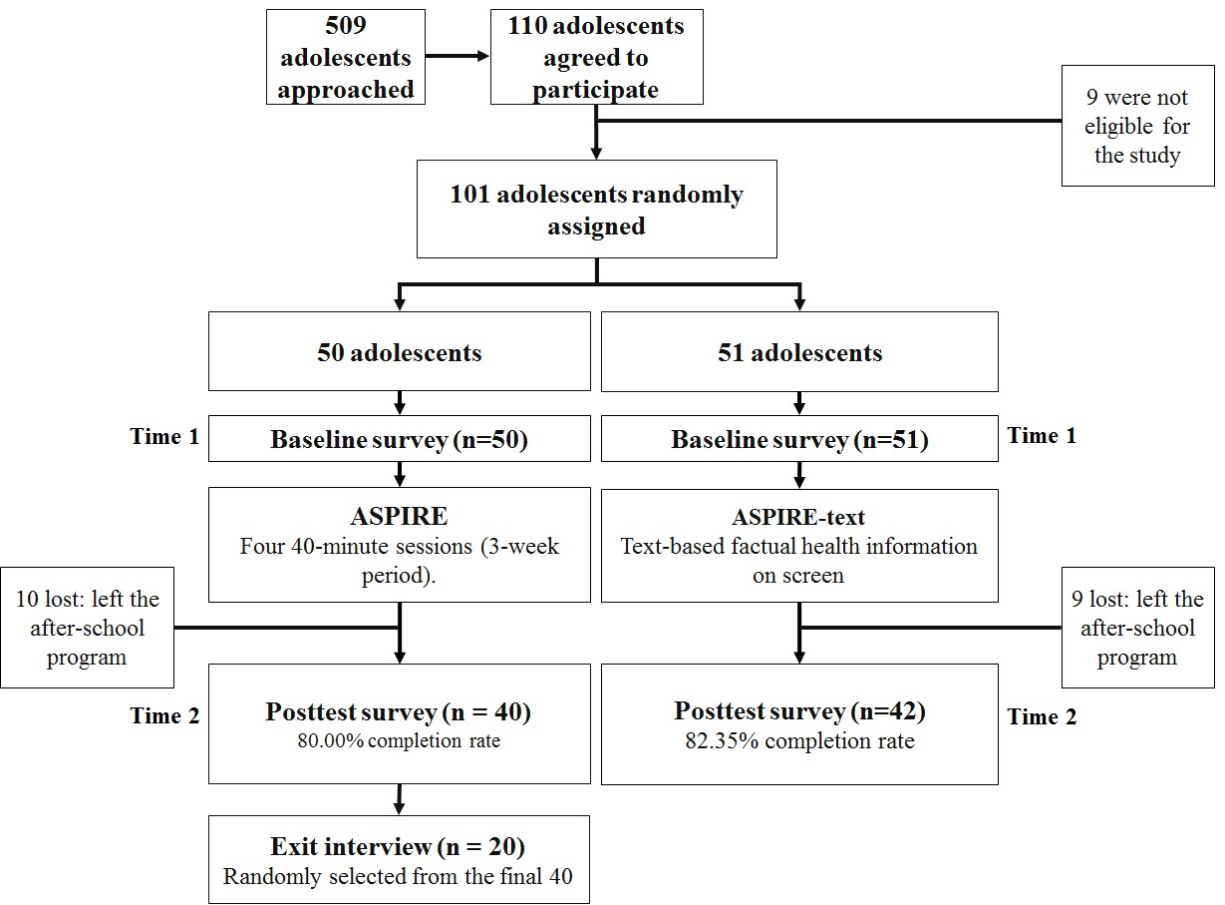
CONSORT (Consolidated Standards of Reporting Trials) flow diagram. The exit interview data are not included in this paper. ASPIRE: A Smoking Prevention Interactive Experience.

A total of 110 adolescents agreed to participate. We excluded 9 adolescents who did not meet the adolescent age criterion (ages 12 through 18 years). A total of 101 participants took the baseline survey and were randomized to 1 of the 2 conditions. All 101 participants went through ASPIRE and ASPIRE-control as prescribed and completed all sessions. Then, 81.20% (82/101) continued until follow-up (81.2% completion rate; Figure 2). Participants who did not continue were adolescents who have left their after-school program.

There was no significant difference between participants who did and those who did not continue to follow-up with respect to baseline intention to smoke (*F*_1,99_=0.03, *P*=.87), prior knowledge about smoking outcomes (*F*_1,100_=0.13, *P*=.72), number of friends who smoke (*F*_1,94_=0.09, *P*=.76), number of detentions at school (*F*_1,95_=0.70, *P*=.40), age (*F*_1,99_=1.15, *P*=.29), gender (χ^2^_1_=0.03, *P*=.85), ethnicity (χ^2^_1_=0.5 *P*=.50), prior frequency of Internet use (*F*_1,96_=2.72, *P*=.10), and skills in Internet use (*F*_1,100_=1.76, *P*=.19).

### Manipulation Checks

#### Perceived Credibility of Health Information

We checked to make sure that the conditions present credible health messages. As expected, there was no significant difference between the conditions with regard to perceived credibility of message content, *F*_1,56_=3.67, *P*=.06. Perceived credibility scores for both groups were found to be significantly greater than 3 on a 5-point Likert scale (ASPIRE: *t*_40_=4.38, *P*<.001; control: *t*_42_=8.48, *P*<.001). This confirms that both interventions were perceived to be credible sources of information related to smoking.

#### Checking for Baseline Differences

There was a marginal difference between the groups with respect to age, ethnicity, and number of friends who smoke (Table 3). When controlled, these differences did not affect the results of this study. The 2 groups did not differ with respect to the number of school detentions, prior knowledge about the effects of smoking, or baseline level of intention to smoke (Table 3).

#### Checking for the Digital Divide

The results indicate that the 2 groups did not differ with respect to the frequency of Internet use in hours per day (Table 3). There were no differences between ethnicities (*F*_1,93_=0.12, *P*=.73), genders (*F*_1,96_=0.60, *P*=.44), those who have and those who have not had detentions (*F*_1,93_=1.32, *P*=.25), or those who have and those who have not had friends who smoke (*F*_1,91_=3.30, *P*=.07), with respect to the frequency of Internet use.

### Checking for Confounders

To check for potential demographic confounders, we determined whether intervention effects varied by demographic characteristics, using moderation analysis with mixed-effects models. Overall, the results failed to identify differential effects as a function of age (*P*=.11), ethnicity (*P*=.43), number of detentions (*P*=.55), frequency of Internet use (*P*=.39), knowledge about smoking effects (*P*=.36), or number of friends who smoke (*P*=.76). Being male (*P*=.01) and having higher grades (*P*=.02) moderated the effect of ASPIRE on intention to smoke.

### From Design Manipulation to User Experience

To test hypothesis 1, we checked to see if the manipulation of interactivity and entertainment has led to an experience of interactivity and entertainment among adolescent users. There was a significant difference between the ASPIRE group and the ASPIRE-control group with respect to perceived interactivity (*F*_1,82_=11.66, *P*=.001) and perceived entertainment (*F*_1,81_=16.40, *P*<.001).

### Intention to Smoke

**Figure 3:**
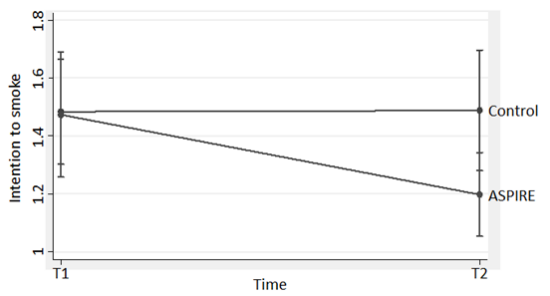
Change in intention to smoke over time for ASPIRE (A Smoking Prevention Interactive Experience) and ASPIRE-control.

A mixed-effects model predicting intention to smoke and controlling for confounders showed support for hypothesis 2 (Figure 3). Adolescents in the ASPIRE group were significantly more likely to show a decrease in their intention to smoke over time compared with participants in the ASPIRE-control group (group × time interaction effect; beta=−0.18, *P*=.008). The ASPIRE group showed a significant decrease in intention to smoke (slope=−0.28, *P*=.008), while the ASPIRE-control group showed no significant change from baseline to follow-up (slope<0.001, *P*=.99).

### Experience of Interactivity and Entertainment

There was a significant correlation between perceived interactivity and perceived entertainment (*r*=.68, *P*<.001). This correlation is expected, considering that interactivity and entertainment in ASPIRE overlap through user activities. Supporting hypothesis 3, mixed-effects models of Table 4 showed that perceived interactivity and perceived entertainment independently worked as significant predictors of a decrease in intention to smoke (model 1: beta_interactivity_=−0.27, *P*=.004 and model 2: beta_entertainment_=−0.20, *P*=.04). When both factors are included in the model (model 3), only perceived interactivity significantly predicted a decrease in intention to smoke (model 3: beta_interactivity_=−0.23, *P*=.04; beta_entertainment_=−0.06, *P*=.61). In all 3 models, the group-by-time interaction effect maintained significance (Table 4).

**Table 4:** Repeated-measures mixed-effects models with perceived interactivity and perceived entertainment predicting intention to smoke.

Variables	Intention to smoke^a^					
	Model 1		Model 2		Model 3	
	beta (SE)^b^	*P* value	beta (SE)	*P* value	beta (SE)	*P* value
Perceived interactivity^c^	−0.27 (0.01)	.004	-	.039	-0.23 (0.01)	
Perceived entertainment^c^	-		−0.20 (0.01)	.038	−0.06 (0.01)	.60
Condition	0.11 (0.17)	.355	0.10 (0.18)	.462	0.16 (0.18)	.37
Time	0.01 (0.06)	.797	0.01 (0.06)	.856	0.01 (0.06)	.85
Condition × time^c^	−0.22 (0.11)	.007	−0.19 (0.11)	.007	−0.19 (0.11)	.007
Intercept	3.05 (0.81)	<.001	2.92 (0.83)	<.001	3.16 (0.89)	<.001
Wald chi-square	34.32	<.001	35.04	<.001	36.68	<.001

^a^Indicates dependent outcome variable.

^b^Indicates standardized values followed by standard error.

^c^Indicates variables of interest. The models control for covariates (age, gender, prior knowledge, school grades, school detentions, and number of friends who smoke), with no significant relationship between such covariates and intention to smoke.

### Path Models

**Figure 4:**
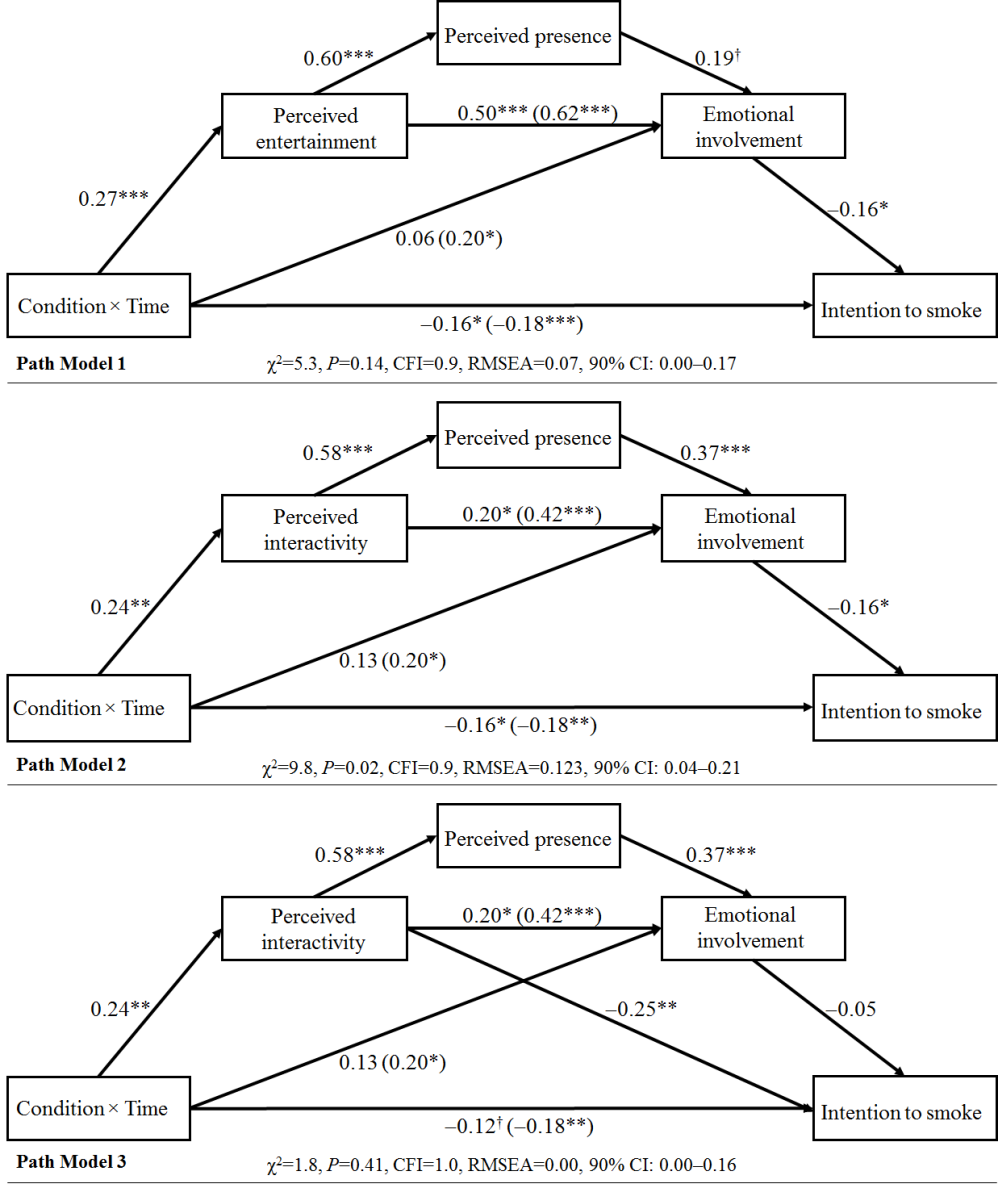
Path models indicating the path from ASPIRE (A Smoking Prevention Interactive Experience) use to intention to smoke. Note. By controlling for the effect of confounders and demographic group differences at baseline, the results remained the same. CFI: comparative fit index; RMSEA: root mean square error of approximation * *P*<0.05; ** *P*<0.01; *** *P*<0.001; † *P*<0.1.

Controlling for baseline group differences and confounders, path model results remained the same. With perceived entertainment (path model 1, Figure 4), path analysis showed that adolescents in the ASPIRE condition were more likely to experience entertainment than adolescents in the control group (beta=0.27, *P*<.001). The relationship between ASPIRE use and emotional involvement was lost when perceived entertainment was added to the model (beta=0.06, *P*=.40). Perceived entertainment fully mediated this relationship, indicating support for hypothesis 4. In model 1, perceived entertainment was significantly related to emotional involvement (beta=0.50, *P*<.001). This relationship remained when perceived presence was added to the model (beta=0.62, *P*<.001). Also, perceived presence was not related to emotional involvement (beta=0.19, *P*=.05). Unsupportive of hypothesis 5, perceived presence did not mediate the relationship between perceived entertainment and emotional involvement. Following this path in model 1, emotional involvement was related to lower intention to smoke (beta=−0.16, *P*=.01; hypothesis 7). Overall, model 1 presented acceptable fit when predicting intention to smoke (χ^2^_3_=5.3, *P*=.142, CFI=.9, RMSEA=.07, 90% CI .00-.17). The model explained 9.7% of the variance in intention to smoke (coefficient of determination, CD=0.097). There were no modification indices needed to improve this model.

With perceived interactivity (path model 2, Figure 4), path analysis showed that adolescents in the ASPIRE condition were more likely to experience interactivity than adolescents in the control group (beta=0.24, *P*=.003). The relationship between ASPIRE use and emotional involvement was lost when perceived interactivity was added to the model (beta=0.13, *P*=.10), indicating full mediation that supports hypothesis 4. The relationship between perceived interactivity and emotional involvement was weakened when perceived presence was added to the model as a mediator (beta=0.20, *P*=.01). Testing for hypothesis 6, perceived presence partially mediated the relationship between the experience of interactivity and emotional involvement. Following this mediation, emotional involvement was related to lower intention to smoke (beta=−0.16, *P*=.01; hypothesis 7). Overall, the model offered poor fit predicting intention to smoke (χ^2^_3_=9.8, *P*=.02, CFI=.9, RMSEA=.123, 90% CI .04-.21). The model explained 9.90% of the variance in intention to smoke (CD=0.099). Modification indices called for the addition of a causal path from perceived interactivity to intention to smoke. As a result, model 3 was constructed with this addition.

Path model 3 of Figure 4 indicated a significant direct relationship between perceived interactivity and intention to smoke (beta=−0.25, *P*<.001). The relationship between emotional involvement and intention to smoke lost significance as a result of this addition (beta=−0.05, *P*=.50). All other paths kept significance. Model 3 indicated acceptable fit when predicting intention to smoke (χ^2^_2_=1.8, *P*=.41, CFI=1.0, RMSEA=.00, 90% CI .00-.16). The model explained 9.0% of the variance in intention to smoke (CD=0.090). There were no modification indices needed to improve this model.

## Discussion

### Conclusions

This post hoc study of a randomized controlled trial examined the process by which adolescents’ experience with a Web-based smoking prevention intervention leads to a health outcome. This study was the first step to better understand the underlying mechanism of eHealth effects, with a user-centered approach. The study identified salient variables in this mechanism from the perspective of the users’ experience and then postulated and empirically tested a model that can help explain how this mechanism takes place cognitively and emotionally, to reach the intended health outcome.

In particular, while ASPIRE has previously shown success in delaying smoking initiation [19], this study showed how adolescents’ experience with ASPIRE contributes to expected outcomes such as decreased intention to smoke. The findings expand previous efforts to explain the effects of Web-based interventions. They (1) go beyond focus on the health outcomes and (2) explicate associations that allow emotional involvement to be a driver of smoking prevention.

The results demonstrate the advantages of a more engaging user experience perceived as interactive and entertaining over the mere exposure to health information in conventional interventions. The 2 critical elements of the user experience manipulated in this study are interactivity and entertainment, as ASPIRE features activities with real-time feedback and entertaining videos. As expected, such manipulation predicted a perception of interactivity and entertainment. The more adolescents found ASPIRE to be interactive and entertaining, the more they were likely to show a decrease in their intention to smoke. A closer look at these findings shows that perceived interactivity in ASPIRE has a stronger relationship with the decrease in intention to smoke than perceived entertainment. This may not necessarily be due to the lack of entertainment in ASPIRE. Instead, this finding may be due to adolescents’ tendency to prefer first-hand involvement in activities and experiential learning instead of exposure to entertaining videos. As a result, Web-based smoking prevention programs that use entertainment need to concentrate their efforts on interactive elements that can boost the effect of entertainment on adolescents’ intention to smoke.

The results also suggest that adolescents tend to transition from the experience of entertainment and interactivity to a psychological state of reduced intention to smoke, passing through emotional involvement. Adolescents transitioned from the experience of media elements (interactivity and entertainment) to perceived presence, emotional involvement, and ultimately lower intention to smoke. This model suggests that the elements of Web-based interventions for smoking prevention (eg, interactivity and entertainment) can have specific psychological effects beyond those explained by cognitive theories (eg, the social learning theory and the health belief model). In particular, concepts such as perceived presence and emotional involvement contribute to predicting tobacco control outcomes.

Furthermore, perceived interactivity and perceived entertainment took separate paths to reach emotional involvement in ASPIRE. Perceived interactivity did not directly correlate with a state of emotional involvement in ASPIRE. Instead, perceived presence allowed for an indirect relationship between perceived interactivity and emotional involvement. The more adolescents found ASPIRE to be interactive, the more they felt present in the ASPIRE environment. Then, perceived presence drove emotional involvement in ASPIRE. On the other hand, perceived entertainment exhibited a direct relationship with emotional involvement, consistent with previous research that presents entertainment as a driver of emotions through drama [88], humor [89], and horror [90].

The findings support the notion that emotional involvement is important if we are to impact adolescent intentions. Emotional involvement seems to play a role in ASPIRE by bridging the gap between experience and smoking intentions. In addition to user activities, the videos in ASPIRE can have a strong emotional influence. Such videos portray dramatic stories through testimonials from smokers facing the effects of smoking, humoristic stories about social situations and smoking outcomes, and fear appeal through the depiction of oral and maxillofacial cancer as a result of smoking. Regardless of the user experience, entertainment and interactivity seem to elicit emotions that predict a decrease in intention to smoke. This supports previous work suggesting that messages with an emotional tone have an impact on youth smoking behavior [91,92]. Future research can expand this work to explore the psychological processes that move adolescents from emotional involvement to lower intention to smoke. One unexpected finding is the enhancement of path model 2 through the addition of a direct relationship between perceived interactivity and intention to smoke. Added in path model 3, this relationship rendered the effect of emotional involvement on intention to smoke nonsignificant. This result corroborates the important role of interactivity in driving health outcomes.

### Limitations

Some limitations for this study must be noted. Participants in both conditions were asked to sit in front of laptops and follow a stringent regimen of ASPIRE usage. During each session, they had to keep an unchanged sitting position without peer-to-peer interaction. While this procedure did not allow adolescents to behave as in a natural environment, it created a uniform Web-based experience that controls for any contamination of results that may be due to distractions during intervention use.

Although the study design involved a manipulation of ASPIRE that mechanically removed entertainment and interactivity from the intervention to create the control condition, the study design did not separate interactivity from entertainment. ASPIRE was designed in a Flash Player format, which is costly to manipulate. In addition, several activities in ASPIRE involved an amalgam between interactivity and entertainment. As a result, the separation between these features in ASPIRE is not sound because it can disrupt the overall ASPIRE experience. In the future and outside the context of ASPIRE, we plan to conduct a study that physically separates interactivity from entertainment and compares the 2 features with each other.

The findings of the transitional model must be interpreted with caution, considering that data analysis is conducted through regression and causation cannot be inferred (eg, change over time in emotional involvement). Nevertheless, path analysis supports the presence of transition that can be confirmed in the future, through a causation model.

Although the study predicted intention to smoke, it did not consider long-term opportunities for protective behaviors or the measurement of actual smoking initiation. While smoking initiation was already examined during the main randomized controlled trial for ASPIRE [19], this study was meant to study the role of adolescents’ experience with ASPIRE in driving smoking-related outcomes. Also, intention to smoke is the closest, most potent predictor of smoking initiation [59,60].

Finally, it must be noted that the opportunity to provide a deep and comprehensive analysis is limited by the relatively small sample size. Future work may consider examining how the ASPIRE experience can prevent long-term smoking initiation with a larger sample of adolescents.

### Implications

Several implications for future research and practice can be envisioned. The results of this study indicate that researchers can work to enhance the experience of interactivity and entertainment in order to better reach outcomes related to nicotine and tobacco control. First, the study of intervention experience can ultimately inform new ways to improve content of Web-based smoking prevention interventions. Through a collaboration with health communication scientists, public health researchers can work to maximize emotional involvement through the elements of interactivity and entertainment. Second, the results encourage further investigation of entertainment in order to find ways to improve its impact. Knowing that interactivity plays an important role in reducing intention to smoke, intervention designers can make use of entertaining features in an interactive environment. For instance, ASPIRE researchers may introduce game-based activities into their interventions. By incorporating game elements into purely interactive activities, adolescents may be transported into a playful environment that can increase their emotional involvement [93,94]. In the future, we plan to examine further how adolescents go from a state of emotional involvement to a decrease in intention to smoke. We plan to also examine how negative and positive emotions and discrete emotions (eg, happiness, sadness, surprise, and fear) may differ in their effects on intention to smoke. We also plan to study further the threshold for entertainment and interactivity required for the program to effectively influence intention to smoke. Finally, future endeavors may involve the application of the user experience model in the context of other health behaviors in order to test its generalizability.

## Appendix

Multimedia Appendix 1 Screenshots of the ASPIRE (A Smoking Prevention Interactive Experience) and ASPIRE-control webpages.

Multimedia Appendix 2 Layout and design of A Smoking Prevention Interactive Experience (ASPIRE).

Multimedia Appendix 3 A video of the ASPIRE (A Smoking Prevention Interactive Experience) website presenting an activity in action.

Multimedia Appendix 4 Checklist for Reporting Results of Internet E-Surveys (CHERRIES).

Multimedia Appendix 5 CONSORT eHealth V1.6.1 checklist.

## Abbreviations

ANOVA: analysis of variance
ASPIRE: A Smoking Prevention Interactive Experience
CD: coefficient of determination
CFI: comparative fit index
CHERRIES: Checklist for Reporting Results of Internet E-Surveys
CONSORT: Consolidated Standards of Reporting Trials
CONSORT-EHEALTH: Consolidated Standards of Reporting Trials of Electronic and Mobile Health Applications and Online Telehealth
E-ELM: extended-elaboration likelihood model
ELT: experiential learning theory
RMSEA: root mean square error of approximation
YMCA: Young Men's Christian Association
