# Supplementary figures and images for: From the Experience of Interactivity and Entertainment to Lower Intention to Smoke: A Randomized Controlled Trial and Path Analysis of a Web-Based Smoking Prevention Program for Adolescents
Source: J Med Internet Res. 2017 Feb 16;19(2):e44. doi: 10.2196/jmir.7174 (PMC5334517; doi:10.2196/jmir.7174)

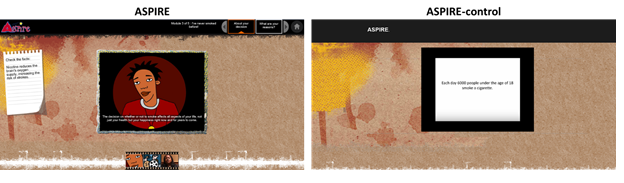

Supplement: Multimedia Appendix 1 [file jmir_v19i2e44_app1.png]

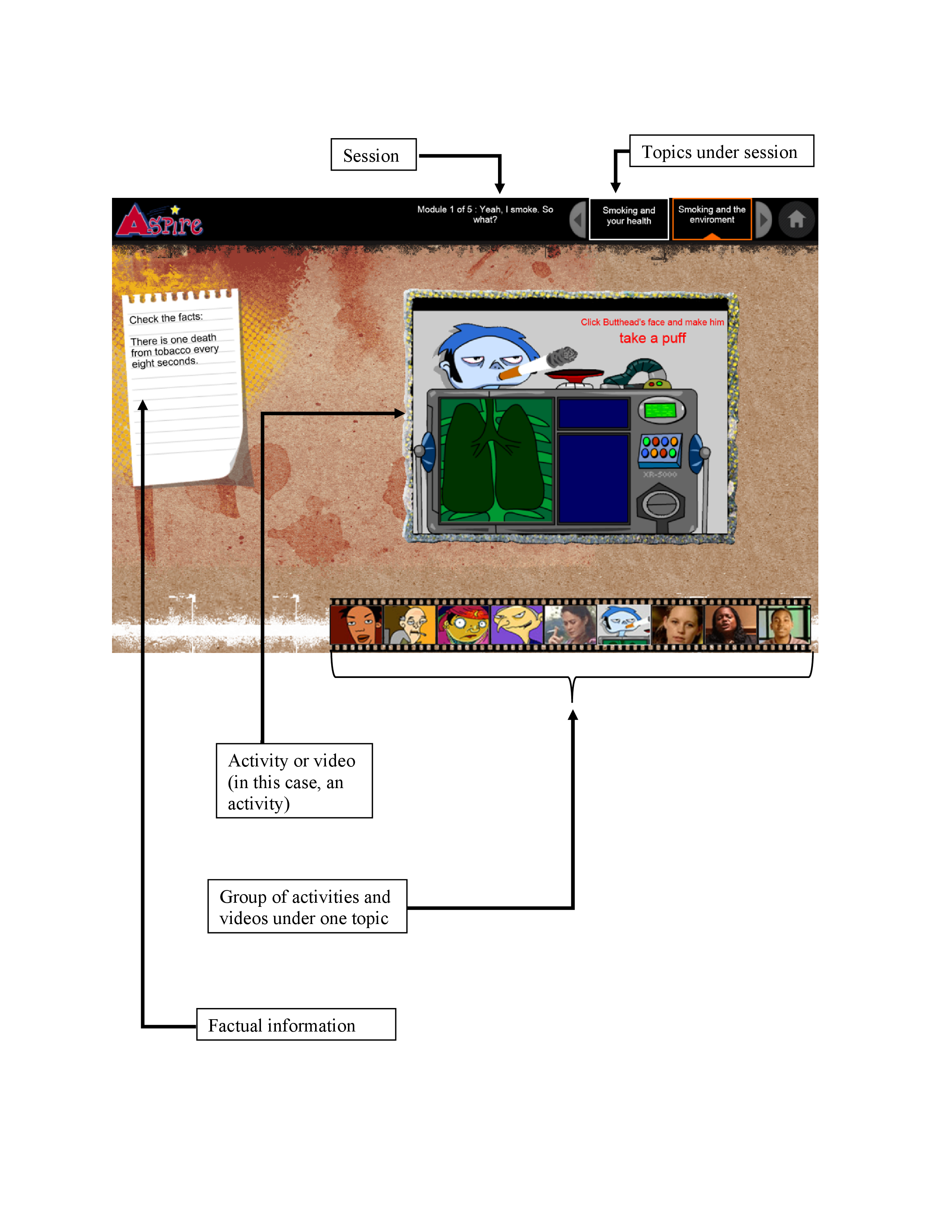

Supplement: Multimedia Appendix 2 [file jmir_v19i2e44_app2.png]
