# Supplementary material for: From the Experience of Interactivity and Entertainment to Lower Intention to Smoke: A Randomized Controlled Trial and Path Analysis of a Web-Based Smoking Prevention Program for Adolescents
Source: J Med Internet Res. 2017 Feb 16;19(2):e44. doi: 10.2196/jmir.7174 (PMC5334517; doi:10.2196/jmir.7174)
Supplement: Multimedia Appendix 4 [file jmir_v19i2e44_app4.pdf]

Checklist for Reporting Results of Internet E-Surveys (CHERRIES)

Design:

The e-surveys of the present study were aimed to collect data from adolescents. The surveys were administered at baseline and post-test (after ASPIRE use).

Development and Pre-testing:

All the surveys were designed using SurveyMonkey (1). The measures were chosen based on previous research in media communication, human-computer communication, and public health (2-17). The surveys were pre-tested for usability and technical functionality during a pilot study. During the pilot study, participants were encouraged to ask questions concerning the surveys and report any concerns to the research team.

IRB Approval, Informed Consent Process, Recruitment, and Survey Administration

The study involving these surveys has been approved by the institutional review board.

The initial contact with potential participants was made face-to-face through an announcement. One month before the experiment, the research team attended the after-school programs in the Houston area, to announce the study and invite participation. The announcement reached as many as 509 adolescents.

The face-to-face announcement explained the purpose of the study, the length of the study, method of accessing the study sign-up sheet, risks/benefits of the study, and statement of voluntary participation.

Interested adolescents completed and signed an assent form and their parents were approached for a parental permission. The baseline survey began with an electronic reminder of consent information. All study surveys were closed to the subsample of adolescents who were interested in the study and who have approved participation along with their parents. The consent form was also provided in hard copy for adolescent participants to keep for their own records. At the experimental site, the consent form was also verbally explained.

For all the surveys, no personal information was collected. All data was retrieved from the cloud and stored in a password protected hard drive and locked in a data storage room. Only the principal investigator and research assistants had access to the data.

Participation in the study was voluntary, and participants were not required to complete the surveys. For completing the surveys and taking part in the intervention, participants were compensated by receiving gift card and give-way items such as school gadgets and toys.

The baseline survey was open for a period of two months. The post-test survey was taken at the intervention site immediately after ASPIRE use. Each survey had between 1 and 13 items per page. The surveys did not allow for the participants to review answers. Participants could not go to a previous page to change their answers.

Response Rate:

Considering the need for full privacy, IP addresses were not collected for participants. Instead, a unique participant was determined based on ID number assigned to them on the first day of the participation. The participation rate for all surveys was 100%. The completion rate for the baseline survey was 100%. The completion rate for the post-test survey was 99%.

Preventing Multiple Entries:

While the surveys were closed, they did not involve a login to prevent duplicate entries. For all surveys, IP addresses were not collected based on the requirements of the institutional review board. However, duplicate database entries having the same ID number were eliminated before analysis, such that the first entry was kept and the second entry was eliminated.

Analysis:

All the data, including incomplete questionnaires, were analyzed. All participants provided answers until the last page of the surveys. No statistical method was needed to correct for missing values.

References:

1. GORDON A. SurveyMonkey. com—Web-Based Survey and Evaluation System: http://www.SurveyMonkey.com, The Internet and Higher Education 2002: 5: 83-87.
2. STRAUB D. M., HILLS N. K., THOMPSON P. J., MOSCICKI A. Effects of pro-and anti-tobacco advertising on nonsmoking adolescents’ intentions to smoke, Journal of Adolescent Health 2003: 32: 36-43.
3. PIERCE J. P., CHOI W. S., GILPIN E. A., FARKAS A. J., MERRITT R. K. Validation of susceptibility as a predictor of which adolescents take up smoking in the United States, Health psychology 1996: 15: 355.
4. CHOI W. S., GILPIN E. A., FARKAS A. J., PIERCE J. P. Determining the probability of future smoking among adolescents, Addiction 2001: 96: 313-323.
5. JACKSON C. Cognitive susceptibility to smoking and initiation of smoking during childhood: a longitudinal study, Preventive medicine 1998: 27: 129-134.
6. CYR D., HEAD M., IVANOV A. Perceived interactivity leading to e-loyalty: Development of a model for cognitive–affective user responses, International journal of human-computer studies 2009: 67: 850-869.
7. NYSVEEN H., PEDERSEN P. E., THORBJØRNSEN H. Intentions to use mobile services: antecedents and cross-service comparisons, Journal of the Academy of Marketing Science 2005: 33: 330-346.
8. SLATER M. D., USOH M., STEED A. Depth of presence in virtual environments, Presence-Teleoperators and Virtual Environments 1994: 3: 130-144.
9. GREEN M. C., BROCK T. C. The role of transportation in the persuasiveness of public narratives, Journal of Personality and Social Psychology 2000: 79: 701-721.
10. HESS U., KLECK R. E. Differentiating emotion elicited and deliberate emotional facial expressions, European Journal of Social Psychology 1990: 20: 369-385.
11. OENEMA A., BRUG J., LECHNER L. Web-based tailored nutrition education: results of a randomized controlled trial, Health education research 2001: 16: 647-660.
12. GRANDPRE J., ALVARO E. M., BURGOON M., MILLER C. H., HALL J. R. Adolescent reactance and anti-smoking campaigns: A theoretical approach, Health Communication 2003: 15: 349-366.
13. ZHAO X., CAI X. The role of ambivalence in college nonsmokers' information seeking and information processing, Communication Research 2008: 35: 298-318.

14. van Deursen A., van Dijk J. Internet skills performance tests: are people ready for eHealth?, Journal of medical Internet research 2011: 13.
15. NationalCancerInstitute D. Monograph 14: Changing Adolescent Smoking Prevalence. Cancer Trends Progress Report: NationalCancerInstitute; 2012.
16. Cokkinides V. E., Halpern M. T., Barbeau E. M., Ward E., Thun M. J. Racial and ethnic disparities in smoking-cessation interventions: analysis of the 2005 National Health Interview Survey, American journal of preventive medicine 2008: 34: 404-412.
17. Anjum Q., Ahmed F., Ashfaq T. Knowledge, attitude and perception of water pipe smoking (Shisha) among adolescents aged 14-19 years, JPMA The Journal of the Pakistan Medical Association 2008: 58: 312.
